# Sociodemographic and geospatial associations with community-associated methicillin-resistant *Staphylococcus aureus* (CA-MRSA) infections in a large Canadian city: an 11 year retrospective study

**DOI:** 10.1186/s12889-019-7169-3

**Published:** 2019-07-09

**Authors:** Victoria C. Gill, Irene Ma, Maggie Guo, Dan B. Gregson, Christopher Naugler, Deirdre L. Church

**Affiliations:** 1 London School of Hygiene and Tropical Medicine, Keppel Street, London, WC1E 7HT UK; 2 Department of Pathology and Laboratory Medicine Cumming School of Medicine, University of Calgary, 3535 Research Rd NW, Calgary, Alberta T2L 2K8 Canada; 3 Department of Medicine, Cumming School of Medicine, University of Calgary, 3330 Hospital Drive NW, Calgary, Alberta T2N 4N1 Canada; 4 Department of Family Medicne, Cumming School of Medicine, University of Calgary, 3330 Hospital Drive NW, Calgary, Alberta T2N 4N1 Canada; 5 Department of Community Health Sciences, Cumming School of Medicine, University of Calgary, 3330 Hospital Drive NW, Calgary, Alberta T2N 4N1 Canada; 6 Alberta Public Laboratories, 3535 Research Road NW, Calgary, Alberta T2L 2K8 Canada

**Keywords:** CA-MRSA, CMRSA10, Geospatial analysis, Laboratory medicine

## Abstract

**Background:**

The first Canadian outbreak of community-associated methicillin-resistant *Staphylococcus aureus* (CA-MRSA) was identified in 2004 in Calgary, Alberta. Using a novel model of MRSA population-based surveillance, sociodemographic risk associations, yearly geospatial dissemination and prevalence of CA-MRSA infections over an 11 year period was identified in an urban healthcare jurisdiction of Calgary.

**Methods:**

Positive MRSA case records, patient demographics and laboratory data were obtained from a centralized Laboratory Information System of Calgary Laboratory Services in Calgary, Alberta, Canada between 2004 and 2014. Public census data was obtained from Statistics Canada, which was used to match with laboratory data and mapped using Geographic Information Systems.

**Results:**

During the study period, 52.5% of positive MRSA infections in Calgary were CA-MRSA cases. The majority were CMRSA10 (USA300) clones (94.1%; *n* = 4255), while the remaining case (*n* = 266) were CMRSA7 (USA400) clones. Period prevalence of CMRSA10 increased from 3.6 cases/100000 population in 2004, to 41.3 cases/100000 population in 2014. Geospatial analysis demonstrated wide dissemination of CMRSA10 annually in the city. Those who are English speaking (RR = 0.05, *p* <  0.0001), identify as visible minority Chinese (RR = 0.09, *p* = 0.0023) or visible minority South Asian (RR = 0.25, *p* = 0.015), and have a high median household income (RR = 0.27, *p* <  0.0001) have a significantly decreased relative risk of CMRSA10 infections.

**Conclusions:**

CMRSA10 prevalence increased between 2004 and 2007, followed by a stabilization of cases by 2014. Certain sociodemographic factors were protective from CMRSA10 infections. The model of MRSA population-surveillance and geomap outbreak events can be used to track the epidemiology of MRSA in any jurisdiction.

**Electronic supplementary material:**

The online version of this article (10.1186/s12889-019-7169-3) contains supplementary material, which is available to authorized users.

## Background

Methicillin-resistant *Staphylococcus aureus* (MRSA) was initially associated with infections acquired in hospital and healthcare environments. In the 1990s, community-associated MRSA (CA-MRSA) cases were reported worldwide [1]. Since then, CA-MRSA has subsequently become the leading manifestation of MRSA infection [2], occurring in many hospital and community settings [1]. Despite having less resistance to other antimicrobial classes than hospital-associated MRSA (HA-MRSA) [3], CA-MRSA has been able to colonize and infect humans effectively, leading to it becoming the dominant *S. aureus* strain in multiple settings [4].

Although there are a variety of CA-MRSA clones, CMRSA10 (USA300) is the predominant cause of skin and soft tissue infection in the United States [4]. The first outbreak of CA-MRSA in Canada was due to the CMRSA10 clone, and was documented in the Calgary Health Region in 2004, when physicians at a local corrections facility noticed an increase in soft tissue infections [5]. A rapid growth in CMRSA10 infections was documented as the number of culture-positive infections climbed from only seven cases between 2002 and 2003, to 42 cases in 2004. It was reported that individuals with a history of illicit drug use, homelessness or recent incarceration were associated with highest risk of infection and accounted for 70% of cases [5].

Community-associated MRSA lacks the traditional risk factors associated with HA-MRSA making identification of those at risk and the monitoring of its spread throughout the general population even more difficult. As the name suggests, CA-MRSA are predominantly acquired in the community, and can spread within families, prisons and sports teams due to close physical contacts (especially those resulting in skin abrasions), the use of shared sanitary facilities, crowded living conditions, poor hygiene, and have been in contact with contaminated objects and surfaces. Transmission of HA-MRSA on the other hand is nosocomial, and its spread is often minimal among household contacts. Patients with CA-MRSA infections are typically younger, and previously healthy when compared to HA-MRSA patients, as patients with HA-MRSA infections are generally elderly, have been hospitalized for prolonged periods, in intensive care units, have indwelling lines or devices, and/or are on long-term antibiotics [1, 2, 6].

Implementation of control strategies for those at highest risk of CA-MRSA in the general population has proven to be challenging. The objective of this study was to use a new model for population-based MRSA surveillance by combining laboratory data, publically available census, and geographic information to identify sociodemographic risk associations, geomap yearly CA-MRSA spread, and document the prevalence of all MRSA cases between 2004 and 2014 within a Canadian healthcare jurisdiction.

## Methods

### Laboratory data and study population

Calgary Laboratory Services (CLS) was created in 1996 and has since then remained the sole diagnostic microbiological testing laboratory in Calgary, Alberta [7], thereby offering an excellent opportunity to investigate the spread of MRSA within a population. Utilizing a comprehensive record of all laboratory results, all positive MRSA cultures from January 1, 2004 to December 31, 2014 were identified. Samples were obtained from patients presenting with symptoms of infection to any Alberta Health Services (AHS) facilities or private health offices including, but not limited to, outpatient clinics, hospitals, general practitioners, and community clinics. Microbiology samples from all sites are processed and analyzed centrally by CLS. All clinical isolates of *S. aureus* were screened and confirmed for the presence of methicillin-resistance according to Clinical and Laboratory Standards Institute antibiotic susceptibility testing guidelines with annual updates to CLS procedures. MRSA positive samples were identified and *mecA* presence confirmed by polymerase chain reaction (PCR), as described previously [8]. During the study period of 2004 to 2010, positive MRSA cultures were routinely typed using pulse-field gel electrophoresis (PFGE) with restriction endonuclease *Sma1,* per standard protocol [9]. From 2010 onwards, *spa* typing was used, as described previously [10].

Records of clinical MRSA cases were retrieved from the CLS database, and any records with missing Provincial Health Numbers (PHN) were excluded from analysis, as the PHN allows for further linkage to obtain the age and gender of each patient. Similar to other studies [11, 12], only the first positive MRSA test result was used in individuals with multiple MRSA test results in any given calendar year, as subsequent MRSA infections have been shown to often be of the original strain of infection in an individual [13]. Inconclusive MRSA cases, and results from all asymptomatic screening tests were excluded from the analysis.

### Census data and geospatial mapping

For the Census years within the study period (2006, 2011), population counts were obtained from Statistics Canada for Calgary [14–16]. For the non-Census years between 2004 and 2014, population estimates were obtained from census population counts from the census years surrounding the study period [14–17], where continuous growth between these values was assumed.

Patient PHN was also used as the linking variable to obtain patient postal codes from an Alberta Health Services database. Postal codes were converted and linked to Statistics Canada’s corresponding census dissemination areas (CDAs) to limit our population to cases within the city of Calgary. Each CDA accounts for 400 to 700 individuals, and are the smallest polygons for which census data are available in Statistics Canada [14], thereby adjusting for the population density. Using ArcGIS (version 10.3), CMRSA10 case maps for each year between 2004 and 2014 were created, as described previously [18]. All potentially identifying information, such as patient PHN and postal codes, were removed for the analysis of the study.

#### Statistical analysis

Period prevalence was calculated to demonstrate the changing burden of all MRSA in Calgary, as the city’s population was collected from Statistics Canada for the census years, and estimated for the non-census years. Due to the infectious nature of MRSA, the entire population of Calgary was considered to be at risk therefore providing our denominator. Summary statistical methods were used to determine the yearly demographics of CA-MRSA infections in Calgary.

A generalized Poisson mixed model was used to examine associations between sociodemographic variables (independent variable) and positive CMRSA10 case rate per CDA (dependent variable) in 2011, as described previously [19–21]. This model was chosen to account for the strata size through the CDAs, where the rate over the CDA was assumed to be constant, and the testing data was independent. Sociodemographic census data was accessed from Statistic Canada’s 2011 Canadian Household Survey for CDA [22], the most recent Census during the study period, where the following sociodemographic variables were included: English speaking, employment rate, education level (having at least some university education), recent immigration status (within the last 5 years), Indigenous status (First Nations, Inuit, and Métis), and the three largest visible minority groups in Canada: ‘Chinese’, ‘South Asian’, and ‘Black’. Median household income (MHI) was also explored and reported for each increase of $100,000 CAD. The reported relative risks (RR) refer to the independent contribution of each sociodemographic variable when compared to its respective reference state. For example, all non-native English speakers from the 2011 census not included in the study was used as a reference state for the “English speaking” sociodemographic variable. *P* <  0.05 was deemed statistically significant (SAS v. 9.4 software).

## Results

**Fig. 1:**
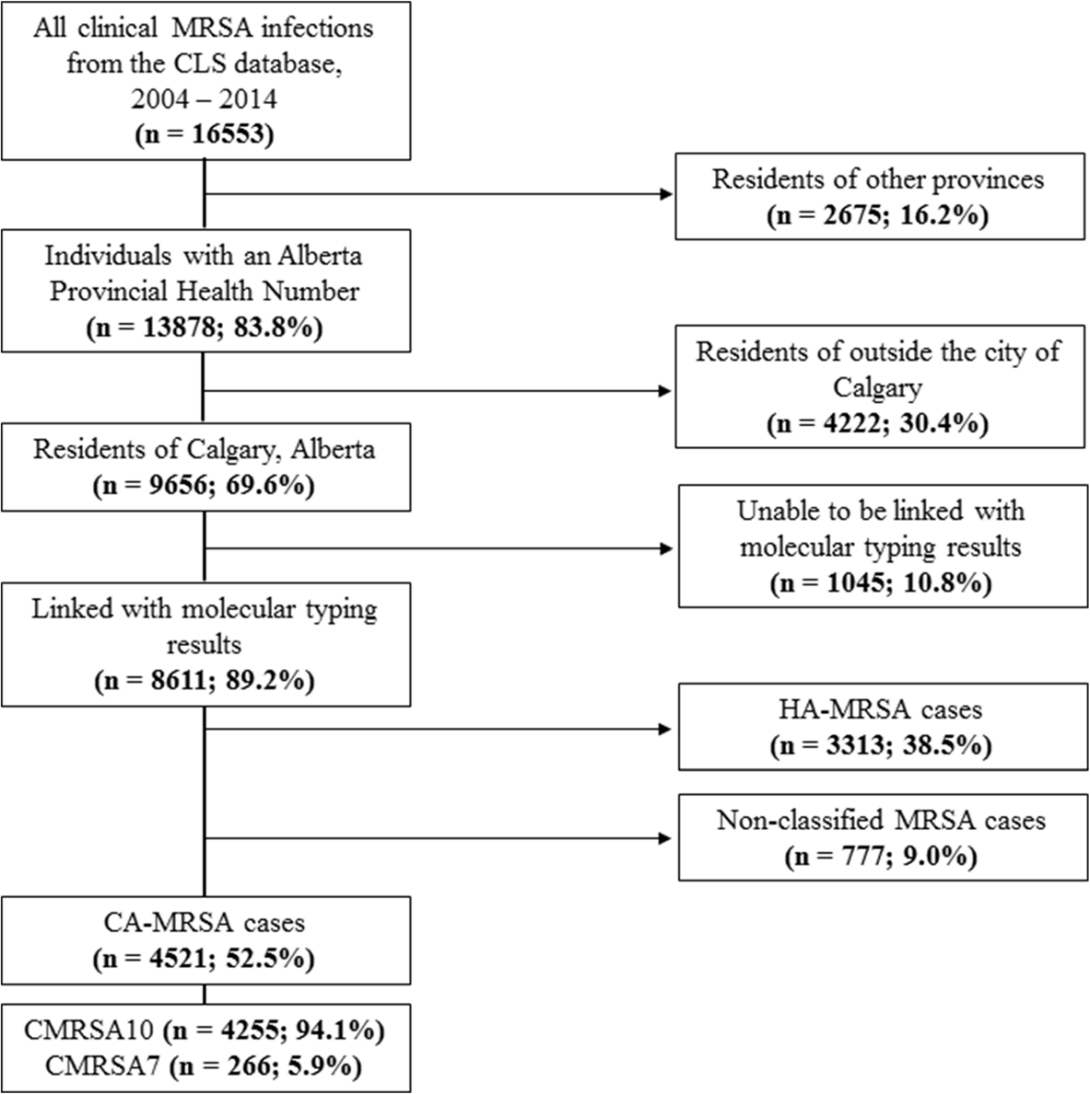
Flow diagram of inclusion (left) and exclusion (right) criteria of study population (2004–2014)

Between January 1, 2004 and December 31, 2014, 16,553 clinical MRSA infections were documented in the CLS database from all sites, including but not limited to community, inpatient, emergency, and outpatient settings. Of these, 13,878 records had an Alberta PHN, which allowed for further linkage to provincial residential postal codes, age, and sex. Residents of the city of Calgary accounted for 9656 of these results, where 8611 of those records were matched with molecular typing results. Community-associated MRSA infections accounted for 4521 of these records, of which, the majority (94.1%) were CMRSA10 cases (Fig. 1).

The prevalence of all MRSA cases in the city of Calgary rose from 22.2 cases/100000 population, to 81 cases/100000 population in 2004 and 2014, respectively. Using Statistics Canada’s census data, CA-MRSA had a prevalence of 4.8 cases/100000 population in 2004, and rose rapidly to 53.19/100000 in 2007, followed by stabilizing to 44.3/100000 by 2014. The prevalence of CMRSA7 (USA400 clone) ranged between 1.2 and 3 cases per 100,000 population during the study period, while CMRSA10 began with a prevalence of 3.6 cases/100000 population in 2004, and increased to 41.3 cases/100000 population in 2014 (Table 1). The median age of all CA-MRSA cases was approximately 41 years, and more than half (*n* = 2802) of the infections were observed in males (Table 2).

**Table 1:** Period prevalence (cases per 100,000 population) of all MRSA cases in Calgary, Canada

Year	Population of Calgary	All MRSA	HA-MRSA	CA-MRSA	CMRSA7 (USA400)	CMRSA10 (USA300)
2004	943,322	22.16	15.27	4.77	1.17	3.60
2005	965,804	46.08	25.16	17.71	1.66	16.05
2006	988812^a^	70.69	35.40	30.64	2.12	28.52
2007	1,009,656	90.53	30.41	53.19	2.28	50.91
2008	1,030,940	84.87	26.58	49.28	2.04	47.24
2009	1,052,672	92.53	31.54	51.77	1.42	50.35
2010	1,074,862	86.80	37.96	44.94	3.16	41.77
2011	1096833^a^	77.31	29.63	40.94	2.37	38.57
2012	1,123,937	77.05	30.07	40.39	2.49	37.90
2013	1,151,712	77.45	24.40	43.67	3.04	40.64
2014	1,180,172	81.01	26.35	44.32	3.05	41.27

^a^Population of Calgary was derived from Statistics Canada’s census data for 2006 and 2011 [15, 17]

**Table 2:** Yearly demographics of community-associated MRSA (CA-MRSA) cases in Calgary, Alberta, Canada

Year	Total number of CA-MRSA cases (n)	Median Age (lower quartile, upper quartile)	Number of Females (%)	Number of Males (%)
2004	45	36.4 (25.5, 50.9)	19 (42.4)	26 (57.8)
2005	171	38.9 (29.3, 47.8)	52 (30.4)	119 (69.6)
2006	303	41.2 (29.4, 51.4)	100 (33.0)	203 (67.0)
2007	537	40.6 (28.0, 50.6)	188 (35.0)	349 (65.0)
2008	508	40.6 (26.9, 51.1)	201 (39.6)	307 (60.4)
2009	545	43.7 (30.2, 54.4)	231 (42.4)	314 (57.6)
2010	483	41.0 (26.7, 55.9)	199 (41.2)	284 (58.8)
2011	449	41.3 (27.8, 55.3)	193 (43.0)	256 (57.0)
2012	454	40.3 (26.5, 57.1)	173 (38.1)	281 (61.9)
2013	503	41.1 (28.2, 54.5)	185 (36.8)	318 (63.2)
2014	523	43.2 (27.9, 56.7)	178 (34.0)	345 (66.0)
Total	4521	40.8 (27.3, 53.6)	1719 (38.0)	2802 (62.0)

**Fig. 2:**
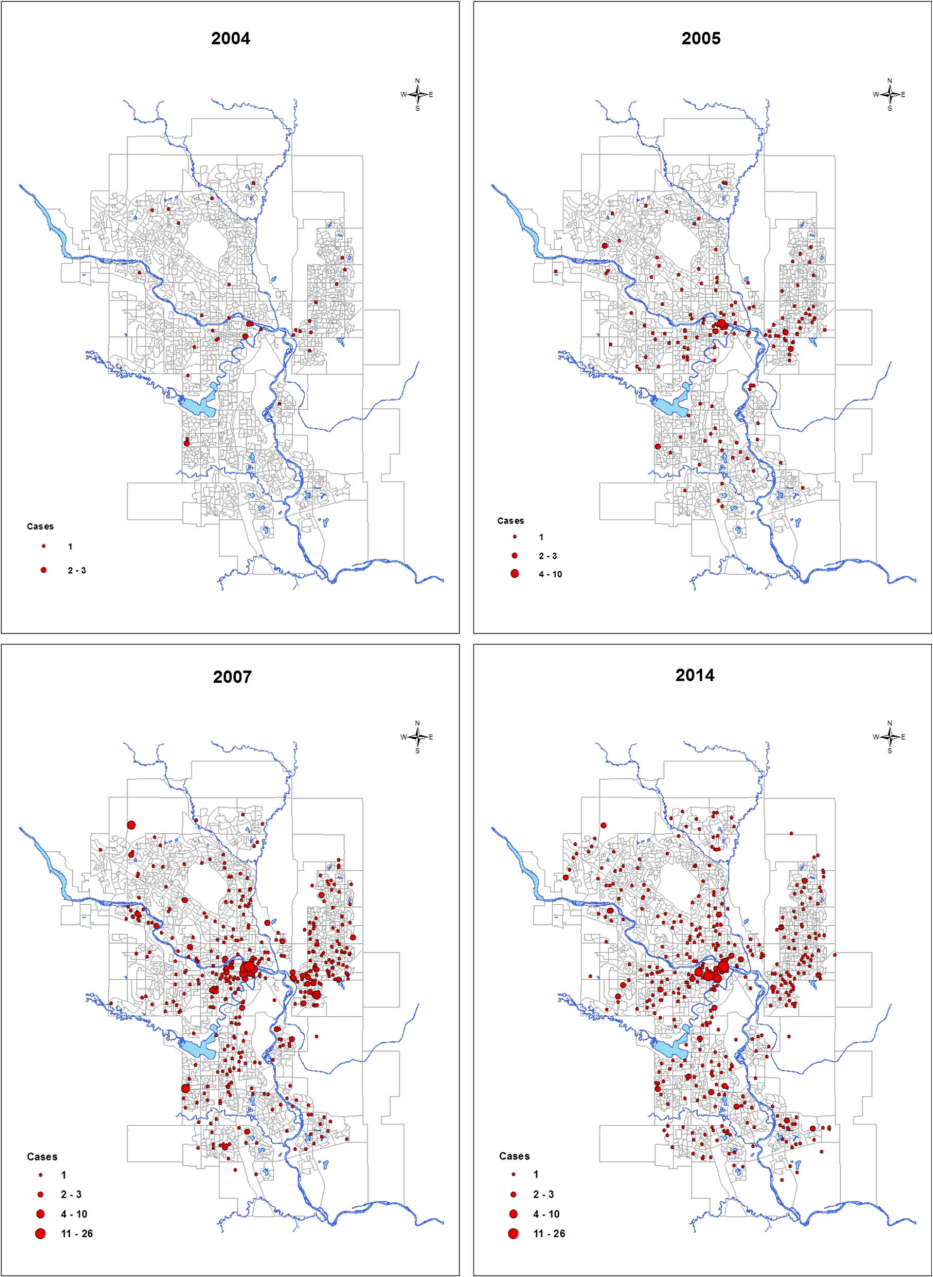
Geospatial dissemination of CMRSA10 cases in Calgary, Alberta during select years of the study period**.** In 2004 (top left), small clusters of CMRSA10 infections in the city of Calgary were already widely disseminated across the city, including the downtown city centre (just south of the Bow River that passes through the city from west to east) and the northern half of the city. Two clusters of up to 3 cases were located both in downtown and the southwest quadrant of the city. In 2005 (top right), the number of cases rapidly increased, spreading in the communities along the Bow River, extending further east and to the southern part of the city. Cases continued to spread and disseminate outwards in all directions as prevalence of CMRSA10 increased in 2007 (bottom left). The downtown core and just east of the Bow River visually appear to be focal regions for CMRSA10 cases, with multiple regions of up to 26 cases per cluster. In 2014 (bottom right), there appeared to be a relative stabilization of positive cases since 2007. For a video of CMRSA10 spread throughout Calgary annually between 2004 and 2014, please see Additional file 1


As 94.1% of CA-MRSA cases were CMRSA10 in Calgary, geographical dissemination and sociodemographic associations were analyzed for this clone. Geospatial analysis revealed that in 2004, CMRSA10 infections in the city of Calgary were already widely disseminated across the city (Fig. 2). In 2005, the number of cases rapidly increased, with regions of up to 10 cases per cluster observed in the downtown region, and spreading in the communities along the Bow River, extending further east and to the southern part of the city. Cases continued to spread and disseminate outwards in all directions as prevalence of CMRSA10 increased in 2007. The downtown core and just east of the Bow River visually appear to be focal regions for CMRSA10 cases, with multiple regions of up to 26 cases per cluster (see Additional file 1 for a video representation of CMRSA10 spread throughout Calgary annually from 2004 to 2014). Although there was a relative stabilization of positive CMRSA10 cases by 2014, there was a substantial increase of clustered cases in the entire downtown region, to a far greater extent than observed in previous years (Fig. 2).

**Additional file 1: Video S1.** Yearly spread of CMRSA10 (USA300) positive cases in the city of Calgary, between 2004 and 2014. (MP4 1200 kb)


A generalized Poisson mixed model revealed that certain sociodemographic variables, such as being an English speaker, of a visible minority Chinese, or of a visible minority South Asian were at a significantly lower risk of CMRSA10 infections in Calgary, Alberta. For every $100,000 CAD increase in median household income, the risk of having a CMRSA10 infection reduces significantly by 73%. All other sociodemographic associations with CMRSA10 infection in Calgary was not statistically significant (Table 3).

**Table 3:** Generalized Poisson mixed model analysis of sociodemographic associations with CMRSA10 cases in Calgary in 2011

Sociodemographic Variables	Risk Ratio (RR)	95% CI	*P-*value
English speaking	0.05	0.01–0.18	< 0.0001
Recent immigrant (within 5 years)	0.65	0.11–3.96	0.6385
Indigenous status (First Nations, Inuit, Métis)	0.61	0.05–7.21	0.6912
Visible minority Chinese	0.09	0.02–0.42	0.0023
Visible minority South Asian	0.25	0.08–0.76	0.015
Visible minority Black	1.03	0.15–6.86	0.9772
Median household income ($100,000 CAD)	0.27	0.19–0.39	< 0.0001
Employment rate	0.45	0.16–1.23	0.1196
Education level (at least some university education)	1.00	0.36–2.72	0.994

## Discussion

By utilizing a novel method of combining laboratory data, publically available census data, and geographic information, the sociodemographic risk associations of the most prevalent type of CA-MRSA, CMRSA10 (USA300 clone), in the general population of over 1.4 million individuals in a Canadian urban healthcare jurisdiction that included community, inpatient and outpatient settings were reported here. An update of the yearly prevalence of all MRSA, and geospatial dissemination of CMRSA10 over an 11 year period since the initial Canadian outbreak in 2004 in Calgary, Alberta, Canada were also reported.

There has been documented decreases of CA-MRSA in some North American populations up to 2011 [23, 24]. However, in this study, the prevalence of CA-MRSA and USA300 infections increased drastically from 2004 to 2007, followed by a relative stabilization with minor fluctuations of prevalence between 2007 and 2014. One possible explanation for the relative stabilization of CA-MRSA infections is the introduction of a formal laboratory surveillance program in Alberta in 2005 after the initial outbreak [25], resulting in increased testing for MRSA infections that normally would not require culturing and could be treated without antibiotic use. As CA-MRSA became increasingly prevalent in our population, this excess culturing may have diminished and resulted in the levelling off to only include clinical infections that require culturing [26]. However, CA-MRSA is still prevalent by the end of the study period, which was expected due to its infectious nature, and investigation into control and prevention strategies are warranted.

Similar to other studies [2, 11], CA-MRSA cases in Calgary, Alberta, affected more males than females, as well as younger individuals. The sex difference observed is unlikely due to an increased biologic susceptibility to CA-MRSA in males, but rather, different exposure to transmission risks between males and females for CA-MRSA, as demonstrated by USA300 affecting more females in a study by Bratu, et al. [27].

Through the analysis of molecular typing results, it became clear that CMRSA10, not CMRSA7, drove the increase of CA-MRSA cases in our population. These results are consistent with studies that find CMRSA10 (USA300) as the predominant clone in urban centres [11, 27], while CMRSA7 (USA400) is the leading clone in rural or remote locations [28, 29]. Some possibilities for the difference in infection rates between the two clones may include competition from CMRSA10 in our population, or different, as yet unidentified, individual and societal risk factors playing a role between clones.

As CMRSA10 was the predominant clone in CA-MRSA cases in Calgary, geospatial mapping and sociodemographic associations were further investigated for this clone. One of the most striking findings from the maps of CMRSA10 cases was the rapid and widespread dissemination across Calgary. This wide distribution of cases over a geographically large area demonstrated that, while CA-MRSA may be acquired in high risk areas, this spread may result in further transmission and reservoirs of infection in other communities. The geographical spread of cases across Calgary may be indicative of hotspots of transmission in areas where large proportions of individuals gather and cross paths (e.g.: in the downtown core), before spreading out across the city. As supported by the initial outbreak of CMRSA10 in 2004 that was connected to those with a history of illicit drug use, homelessness or recent incarceration [5], the clustered cases appear to be focused in the downtown region containing multiple shelters that service those who are homeless or at risk for homelessness, or affected by alcohol and drug dependencies.

Although we are not able to draw inferences of multiple combinations of sociodemographic associations with positive CMRSA10 case rate, as each variable was analyzed as an independent contribution, our model demonstrated that certain sociodemographic variables, such as English speaking, being of visible minority Chinese or South Asian, or having a high median household income, were associated with a decreased risk of acquiring CMRSA10. The relationship between income and disease risk was as predicted as increased income is commonly associated with improved health, living conditions and decreased residential crowding, which would likely limit the risk of CA-MRSA transmission [2, 30]. Risk factors such as homelessness and incarceration, as documented in 2004 in our population, are expected to decrease with increased income.

Those who identify themselves as native English speakers in the Census also demonstrated a decreased risk for acquiring CMRSA10 compared to those who do not identify as native English speakers. One reason could be attributed to the ability for the patient to understand promoted behaviours and health interventions that are aimed at reducing risk by healthcare providers [31].

Visible minority Chinese status and visible minority South Asian status were variables that presented with decreased risk of CMRSA10. We did not expect to see any evidence in these variables reflecting either increased, or decreased risk. It is unlikely that these variables were protective factors against CMRSA10, but was a surrogate marker for a variable that were unmeasured.

Although studies have shown that Indigenous populations [1, 32, 33] and African-Americans [27, 34] are more likely to be at risk for CA-MRSA infections, our analysis did not show significant associations between CMRSA10 cases with Indigenous or the visible minority Black populations of Calgary. However, it has been shown that Canadian Indigenous populations are more likely associated with CMRSA7 clone, rather than CMRSA10 [28, 35, 36]. As there is no biological plausibility for certain ethnicities to be at an increased risk for CA-MRSA infections, this may be reflective of larger societal differences between our population and those in other studies.

### Limitations

As routine culture is not recommended in current guidelines [26], along with the lack of population screening strategies, only patients with microbiologically confirmed infections were included, while asymptomatic carriers were excluded in our analysis, which may have resulted in an underestimation of the true prevalence of CA-MRSA infection within Calgary. Furthermore, as any patient without a Provincial Health Number were excluded in our study analysis (temporary workers and visitors from outside of Alberta), this may have also contributed to the underestimation of CA-MRSA prevalence within the city of Calgary. Although this proportion would likely be small, the patients excluded from our study criteria receiving acute medical care in Calgary would represent an important population regarding the spread and transmission of CA-MRSA. Despite these limitations, the data likely closely reflects the true prevalence of more serious CA-MRSA infections where routine culture is strongly recommended in guidelines [26].

Another limitation of this project was that the data obtained and analysis performed were at the population level. The ecological fallacy that what holds true at a population level also holds true at an individual level mandates cautious interpretation of our results. While our data appear to support the association of documented individual risk factors with CA-MRSA infection, we cannot confirm that these factors are the cause of what we are observing nor extrapolate these individual risk factors to communities in other jurisdictions.

The system that we used to obtain the residential data used the listed residence at fiscal year-end as the location of record, thus the case location did not necessarily indicate where CMRSA10 infections were acquired, nor does it indicate the residence at the time of infection if the individual moved within the calendar year. The spread of cases observed from our maps could be different from the locations where risk factors are, and where transmission is occurring. Despite the limitations they still provide valuable insight to the spread and distribution of CMRSA10 in Calgary over 11 years.

## Conclusions

Although the results of this study focused on an urban healthcare jurisdiction within Canada, the method of combining various databases from Laboratory Information Systems, publically available censuses, and Geographic Information Systems can be utilized in any jurisdiction globally to perform MRSA surveillance and geomap outbreak events. Sociodemographic risk association results from this study can inform healthcare providers to assist in future testing strategies, targeted interventions and control measures in our population. Enhanced efforts should be made to control and prevent CA-MRSA spread.

## Data Availability

The datasets generated during and/or analyzed during the current study are not publically available due to confidentiality and ethics review board restrictions, but are available from the corresponding author on reasonable request.

## Abbreviations

AHS: Alberta Health Services
CA-MRSA: Community-Associated Methicillin-Resistant *Staphylococcus aureus*
CDA: Census dissemination area
CLS: Calgary Laboratory Services
HA-MRSA: Hospital-Associated Methicillin-Resistant *Staphylococcus aureus*
MHI: Median household income
MRSA: Methicillin-Resistant *Staphylococcus aureus*
PCR: Polymerase chain reaction
PHN: Provincial health number
RR: Relative risk
